# Norcantharidin-Encapsulated C60-Modified Nanomicelles: A Potential Approach to Mitigate Cytotoxicity in Renal Cells and Simultaneously Enhance Anti-Tumor Activity in Hepatocellular Carcinoma Cells

**DOI:** 10.3390/molecules28227609

**Published:** 2023-11-15

**Authors:** Zhongpeng Ding, Beihua Xu, Huimin Zhang, Zhenyu Wang, Luying Sun, Mengjie Tang, Meihong Ding, Ting Zhang, Senlin Shi

**Affiliations:** College of Pharmaceutical Sciences, Zhejiang Chinese Medical University, Hangzhou 311400, China; 13839710403@163.com (Z.D.); xvbeihua@163.com (B.X.); zhanghuimin202203@163.com (H.Z.); wzyzcmu@163.com (Z.W.); 15157561337@163.com (L.S.); tang774124625@163.com (M.T.); dingmeihong0125@163.com (M.D.); zhangting55@zcmu.edu.cn (T.Z.)

**Keywords:** norcantharidin, attenuate, C60, nanomicelles, hepatic carcinoma

## Abstract

Objective: The objective of this study was to examine the preparation process of DSPE-PEG-C60/NCTD micelles and assess the impact of fullerenol (C60)-modified micelles on the nephrotoxicity and antitumor activity of NCTD. Method: The micelles containing NCTD were prepared using the ultrasonic method and subsequently optimized and characterized. The cytotoxicity of micelles loaded with NCTD was assessed using the CCK-8 method on human hepatoma cell lines HepG2 and BEL-7402, as well as normal cell lines HK-2 and L02. Acridine orange/ethidium bromide (AO/EB) double staining and flow cytometry were employed to assess the impact of NCTD-loaded micelles on the apoptosis of the HK-2 cells and the HepG2 cells. Additionally, JC-1 fluorescence was utilized to quantify the alterations in mitochondrial membrane potential. The generation of reactive oxygen species (ROS) following micelle treatment was determined through 2′,7′-dichlorofluorescein diacetate (DCFDA) staining. Results: The particle size distribution of the DSPE-PEG-C60/NCTD micelles was determined to be 91.57 nm (PDI = 0.231). The zeta potential of the micelles was found to be −13.8 mV. The encapsulation efficiency was measured to be 91.9%. The in vitro release behavior of the micelles followed the Higuchi equation. Cellular experiments demonstrated a notable decrease in the toxicity of the C60-modified micelles against the HK-2 cells, accompanied by an augmented inhibitory effect on cancer cells. Compared to the free NCTD group, the DSPE-PEG-C60 micelles exhibited a decreased apoptosis rate (12%) for the HK-2 cell line, lower than the apoptosis rate observed in the NCTD group (36%) at an NCTD concentration of 75 μM. The rate of apoptosis in the HepG2 cells exhibited a significant increase (49%), surpassing the apoptosis rate observed in the NCTD group (24%) at a concentration of 150 μM NCTD. The HK-2 cells exhibited a reduction in intracellular ROS and an increase in mitochondrial membrane potential (ΔψM) upon exposure to C60-modified micelles compared to the NCTD group. Conclusions: The DSPE-PEG-C60/NCTD micelles, as prepared in this study, demonstrated the ability to decrease cytotoxicity and ROS levels in normal renal cells (HK-2) in vitro. Additionally, these micelles showed an enhanced antitumor activity against human hepatocellular carcinoma cells (HepG2, BEL-7402).

## 1. Introduction

Norcantharidin (NCTD) is a derivative of Chinese medicine cantharidin (CTD) after the removal of two methyl groups at 1 and 2 positions of cantharidin [1]. NCTD has been shown to inhibit the growth of solid tumors, such as liver cancer, esophageal cancer, gastric cancer, with the advantages of the white blood cell increase, immunity regulation and no bone marrow suppression [2,3,4]. NCTD has been used for many years in China to treat liver cancer and hepatitis through oral administration and injection. It is available in the form of demethylcantharidin tablets and sodium demethylcantharidate for injection.

However, despite its lower toxicity compared to cantharidin, NCTD still involved nephrotoxicity in clinical settings [5]. Following oral administration of a high dose, the glomerular epithelial cells exhibited turbidity and swelling. Toxicology of NCTD in mice found that ROS levels in kidney tissues of mice were significantly increased after the administration of NCTD, and attributable injuries such as tissue congestion and vasodilation occurred [6]. The results of significant toxic effects on normal human cells indicated that NCTD had no selective inhibition of tumors. In addition, the poor solubility, short half-life and low LD50 of NCTD are the disadvantages that limit its clinical application [7].

In order to mitigate the toxic damage to normal cells, we hypothesized that antioxidants could be potential candidates for reducing oxidative stress in cells. This is particularly important due to the high levels of ROS induced by NCTD in normal tissues, which can result in cell injury [6]. It was reported that fullerene, one of the allotropes of the carbon nanomaterial family, had powerful antioxidant properties with long-lasting activity. However, its poor water solubility limits the biological application. Fullerenol, a derivative of fullerene that is soluble in water, showed high ROS scavenging activity both in vivo and in vitro [8,9,10,11]. It has been reported that fullerenol exhibits protective effects against doxorubicin-induced cytotoxicity in the lungs, kidneys and testes of rats [12,13,14,15,16]. In previous experimental studies, we employed micelles that were modified with fullerenol in a drug delivery system that contained doxorubicin hydrochloride (DOX). The findings indicated that the fullerenol (C60)-modified micelles demonstrated reduced cytotoxicity in normal cell lines (L02, H9c2, GES-1) in comparison to free DOX in vitro. The results also showed that micelles modified with C60 exhibited a reduction in intracellular ROS in the H9c2 cells in comparison to free DOX [17]. However, it is still uncertain whether fullerenol has the potential to play a role against the oxidative stress induced by other anticancer drugs in normal tissues.

Polymer micelles have emerged as a promising approach for drug delivery, offering a potential solution to the challenges associated with low bioavailability and poor solubility of free drugs [18]. Polymer micelles are generated through the process of self-assembly involving amphiphilic polymers. Drugs can be either grafted with polymers to produce pharmacologically active polymer systems or encapsulated in the nanoscale-diameter micelles by polymer self-assembly, which provided opportunities for solubility and stability enhancement of the drugs [19,20]. The micelles’ appropriate diameter, typically ranging from 100 to 200 nm, facilitates their accumulation within the tumor microenvironment by leveraging the enhanced permeability and retention (EPR) effects [21,22,23]. Polymerized micelles have demonstrated enhanced pharmacokinetic properties in preclinical animal models, as well as improved therapeutic effectiveness and superior safety. Several polymeric micellar formulations have advanced to the clinical stage, either undergoing clinical trials or having obtained approval for human use [24,25,26]. For instance, regulatory authorities in Korea, China and other countries have granted approval for the use of Genexol^®^ PM, Nanoxel^®^ M, Zicheng^®^ and other drugs as effective treatments for cancer [27,28].

In the present investigation, distearyl phosphacylethanolamine-polyethylene glycol (DSPE-PEG 2000) was used as the skeletal material of the micelles, which was further modified by C60(OH)_22_. DSPE-PEG, which has been approved by the US FDA, is frequently employed for the encapsulation of proteins, peptides and other pharmaceuticals. This utilization aims to extend the half-life of these substances in the bloodstream and enhance their stability. It has also been reported that the utilization of DSPE-PEG as a carrier in a nanodrug delivery system demonstrated enhancements in the cellular uptake and cytotoxicity of insoluble anticancer drugs [29,30]. Hence, this study focused on the preparation of the DSPE-PEG-C60 micelles loaded with NCTD and aimed to investigate their cytotoxicity in both the tumor cells and the normal cells.

## 2. Results

### 2.1. Determination of NCTD by HPLC

**Figure 1:**
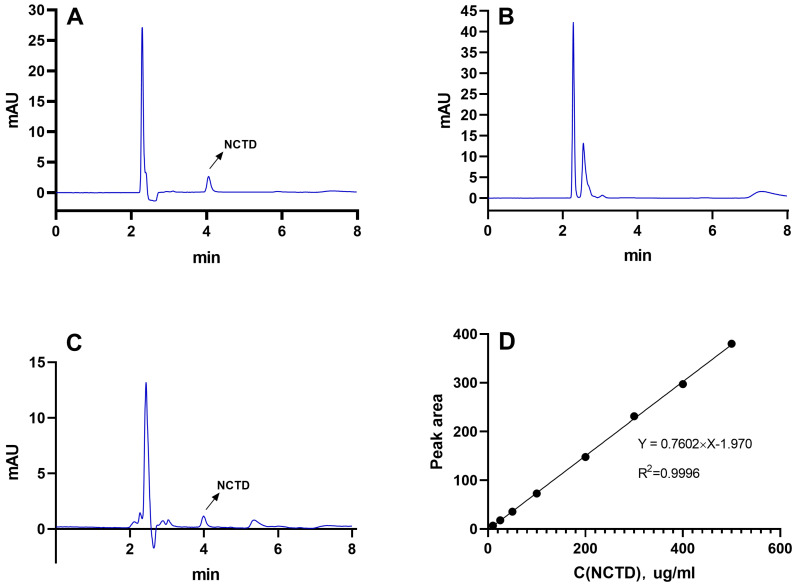
The HPLC spectra of NCTD (**A**), DSPE-PEG-C60 (**B**) and DSPE-PEG-C60/NCTD (**C**) and the standard regression equation curve of NCTD (**D**).

As demonstrated in Figure 1, the retention period of NCTD was approximately 4.1 min (Figure 1A), and DSPE-PEG-C60 did not interfere with NCTD detection (Figure 1B,C). The NCTD regression curve exhibited a solid linear relationship between 59.47 uM and 2973.54 uM (Figure 1D), and the correlation coefficient (R^2^) was larger than 0.9996.

### 2.2. Preparation and Characterization of Micelles

**Figure 2:**
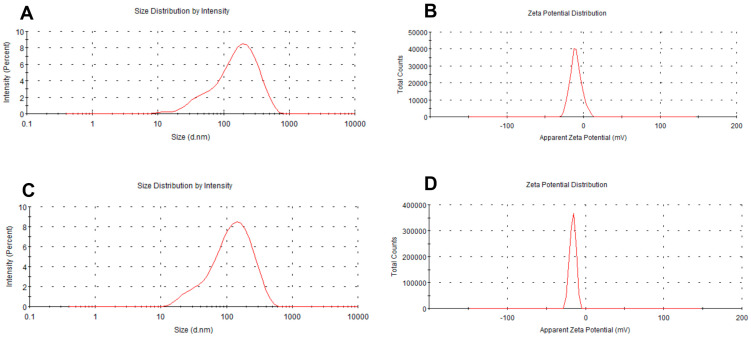
Particle size and zeta potential of micelles: DSPE-PEG/NCTD (**A**,**B**) and DSPE-PEG-C60/NCTD (**C**,**D**).

**Figure 3:**
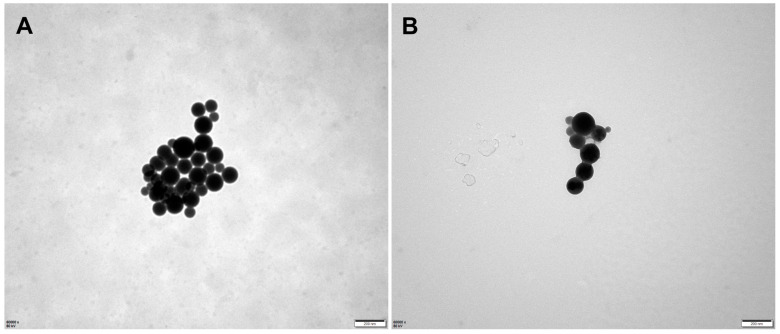
Transmission electron microscopy (TEM) images of micelles: DSPE-PEG/NCTD (**A**) and DSPE-PEG-C60/NCTD (**B**).

The results are presented in Table 1. The sizes of the micelles decreased progressively as the vectors increased. The particle sizes were measured to be 117.8 nm (vector: NCTD = 10:1 (*W*/*W*)), 91.57 nm (vector: NCTD = 15:1 (*W*/*W*)) and 45.74 nm (vector: NCTD = 20:1 (*W*/*W*)) (Table 1, Batches 1, 2 and 3). The findings of the study also demonstrated that the micellar sizes were influenced by the ultrasound time and power. The micelles were able to achieve the desired particle size of about 100 nm in diameter by subjecting them to the ultrasonic treatment for 10 min at an ultrasound power of 200 W. The particle sizes decreased with both an increase and decrease in the ultrasound time and power (Table 1, Batches 2, 4, 5, 6, 7). Finally, the processing parameters that were adopted included an NCTD-to-carrier weight ratio of 1:15, an ultrasound time of 10 min and an ultrasound power of 200 W. The micelles loaded with NCTD exhibited a high encapsulation efficiency (EE) of 91.90% and a drug loading content (LC) of 5.92%. These micelles also demonstrated an ideal particle size distribution of 91.6 nm and a negative charge of −13.8 mV. The unmodified DSPE-PEG micelles were also prepared using the same process, with a similar particle size of 96.1 nm, as shown in Table 2 and Figure 2. TEM images depicting the micelles are presented in Figure 3.

Compared to the results obtained from DLS, the particle size appeared smaller in TEM detection. This is because the micelles undergo shrinkage during the drying process, leading to a reduction in particle size. In dynamic light scattering (DLS), however, micelles form hydration layers with surrounding water molecules, which leads to larger results in particle size detection using DLS.

### 2.3. Stability of Micellar Solution

**Figure 4:**
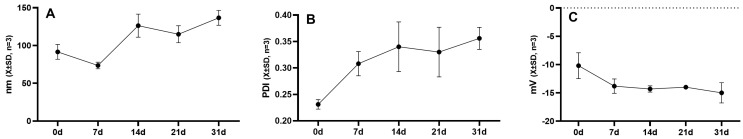
Changes in particle size (**A**), PDI (**B**) and zeta potential (**C**) of DSPE-PEG-C60/NCTD micelles at 4 °C.

The impact of temperature on the stability of the micellar solution is demonstrated in Figure 4. The micellar solution of DSPE-PEG-C60/NCTD exhibited a relatively stable behavior, with minimal variation in particle size over a period of 7 days at a temperature of 4 °C. However, the turbid phenomenon of DSPE-PEG-C60/NCTD micellar solution was observed at room temperature during the experimental process.

### 2.4. Drug Release Assay

**Figure 5:**
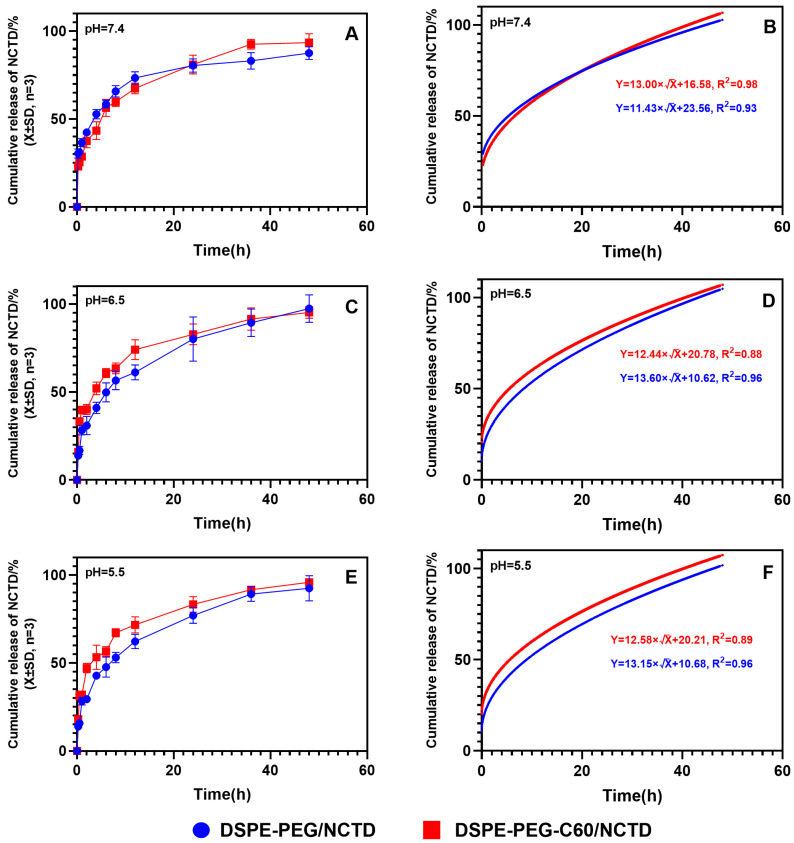
The cumulative release kinetics of NCTD from micelles and the Higuchi equation. Cumulative release kinetics and Higuchi equation of NCTD micelles at different pH: pH = 7.4 (**A**,**B**), pH = 6.5 (**C**,**D**) and pH = 5.5 (**E**,**F**).

As depicted in Figure 5, the release of the DSPE-PEG-C60/NCTD and DSPE-PEG/NCTD micelles in PBS buffers (including 1% SDS) followed the Higuchi equation. After a duration of 6 h, the cumulative release rates of NCTD from the DSPE-PEG-C60/NCTD micelles and the DSPE-PEG/NCTD micelles were observed to be 56% and 58%, respectively, in pH 7.4 PBS buffers. The in vitro release experiments demonstrated a sustained release of approximately 90% of NCTD over a period of 48 h, with no significant burst release observed (Figure 5A). In line with our earlier research, these findings showed that the introduction of C60(OH)_22_ had no appreciable effect on the micelles’ release kinetics.

Since the pH of tumor tissues is much lower than that of normal tissues, the release profile of NCTD from the micelles was evaluated at pH 5.5 and 6.5 (Figure 5C,E). Approximately 94.3% of the released NCTD was observed from the micelles at pH 6.5, while about 97.4% was observed at pH 5.5 within 48 h. There was no significant difference in the release of NCTD from the micelles in different pH environments. This result suggests that the release of NCTD from micelles is pH-independent.

### 2.5. In Vitro Cytotoxicity Examination

**Figure 6:**
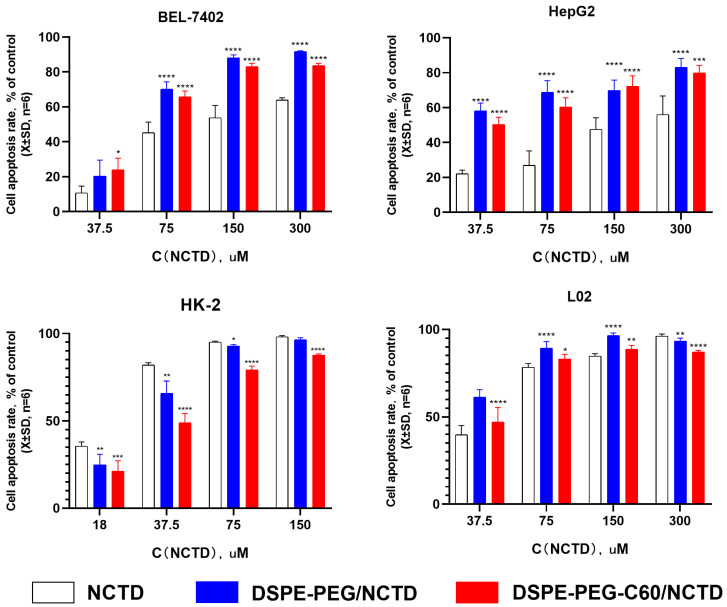
In vitro cytotoxicity of DSPE-PEG-C60/NCTD micelles, DSPE-PEG/NCTD micelles and free NCTD was determined after 72 h using CCK-8 assays to measure cell inhibition rates. Data represent the mean ± SD from three independent experiments (* *p* < 0.05, ** *p* < 0.01, *** *p* < 0.001 and **** *p* < 0.0001).

Cell activity was assessed using the CCK-8 method. The cytotoxicity of the DSPE-PEG-C60/NCTD micelles was evaluated in comparison to free NCTD and the DSPE-PEG/NCTD micelles in the L02, HK-2, HepG2 and BEL-7402 cell lines. As depicted in Figure 6, the encapsulation of NCTD into the micelles resulted in a substantial increase in the inhibition effect on the tumor cells of HepG2 and BEL-7402. The IC_50_ of the DSPE-PEG-C60/NCTD micelles was found to be 90.07 μM in the HepG2 cells and 63.20 μM in the BEL-7402 cells. These values were lower compared to the IC_50_ of the free NCTD group, which was 117.50 μM in the HepG2 cells and 102.62 μM in the BEL-7402 cells. This phenomenon can be attributed to the surface-active properties of the carrier (DSPE-PEG and DSPE-PEG-C60), which can penetrate the cell membrane, inducing the increase in cell membrane fluidity and accelerating the transmembrane turnover of NCTD, contributing to the increased uptake of NCTD by cells and the enhanced cytotoxicity of the NCTD micelles [31,32,33,34].

Interestingly, the micelle groups did not exhibit an elevated cytotoxic effect on the HK-2 cells in comparison to the free NCTD group. The DSPE-PEG-C60/NCTD micelles exhibited less toxicity (IC_50_ = 36.59 μM) than the free NCTD group (IC_50_ = 23.05 μM) on the HK-2 cells. A potential explanation for this observation was that the HK-2 cell line was more susceptible to NCTD than the other cell lines. This increased susceptibility may have compromised the cytotoxicity enhancement capacity of the DSPE-PEG/DSPE-PEG-C60 carrier.

### 2.6. Cell Apoptosis Assay Using Acridine Orange/Ethidium Bromide (AO/EB) Staining

**Figure 7:**
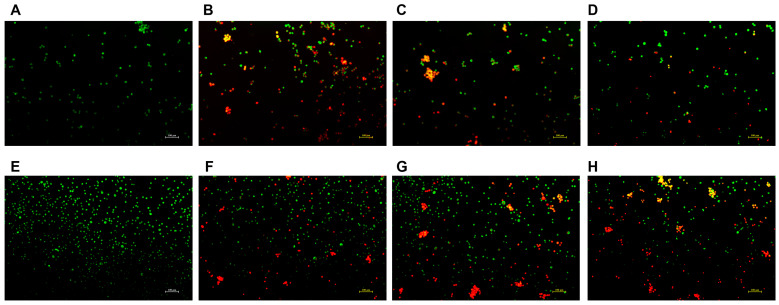
Fluorescence microscopic images showing the live and dead cell assay of NCTD and NCTD-loaded micelles in HK-2 cells and HepG2 cells: control (**A**), free NCTD (**B**), DSPE-PEG/NCTD (**C**), and DSPE-PEG-C60/NCTD (**D**). Cells were treated with micelles or free NCTD at equivalent concentrations of 75 μM NCTD (for HK-2 cells) or 150 μM NCTD (for HepG2 cells) for 24 h. The cells were then stained with acridine orange/ethidium bromide and observed under a fluorescence microscope. (**A**–**D**) shows fluorescence microscopic images of HK-2 cells, (**E**–**H**) shows Fluorescence microscopic images of HepG2 cells. The scale is 100 nm.

Given that hepatocellular carcinoma is the primary clinical context in which NCTD is applied, and considering its significant association with nephrotoxicity, the staining subjects for this study were chosen to be the HepG2 and HK-2 cells. The effect of the DSPE-PEG-C60/NCTD micelles on the cell apoptosis was detected using the AO/EB staining. As depicted in Figure 7, the representative fluorescence microscopic images of the double-stained cells reveal that significant apoptosis was detected in the free NCTD group, whose cells were stained with EB and are shown orange-red in color (Figure 7B,F). The DSPE-PEG-C60/NCTD micelle group showed less orange-red fluorescence than the free NCTD group, indicating a decrease in the cytotoxicity of the DSPE-PEG-C60/NCTD micelles in the HK-2 cell line (Figure 7D). In the case of the HepG2 cells, both the DSPE-PEG/NCTD micelles and the DSPE-PEG-C60/NCTD micelles demonstrated higher induction of apoptotic cells with orange-red fluorescence compared to the free NCTD group (Figure 7G,H).

### 2.7. Mitochondrial Membrane Potential Assay

**Figure 8:**
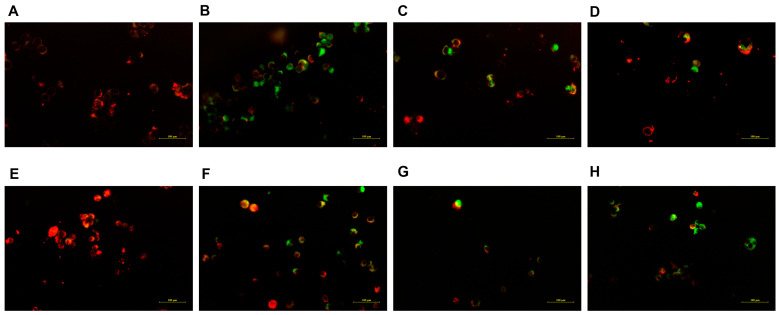
Fluorescence microscopic images showing mitochondrial outer membrane potential (ΔψM) of NCTD and NCTD-loaded micelles in HK-2 cells and HepG2 cells: control (**A**), free NCTD (**B**), DSPE-PEG/NCTD (**C**), and DSPE-PEG-C60/NCTD (**D**). Cells were treated with micelles or free NCTD at equivalent concentrations of 75 μM NCTD (HK-2 cells) or 150 μM NCTD (HepG2 cells) for 24 h with JC-1 staining followed by fluorescence microscope observation. Healthy cells with high ΔψM form JC-1 aggregates showing red fluorescence, while apoptotic cells with low ΔψM exhibit green fluorescence. (**A**–**D**) shows fluorescence microscopic images of HK-2 cells, (**E**–**H**) shows Fluorescence microscopic images of HepG2 cells. The scale is 100 nm.

The functional integrity of mitochondria was evaluated by JC-1 staining. The representative fluorescence microscopic images demonstrate that the administration of free NCTD resulted in a reduction in the number of JC-1 aggregates (red fluorescence) and an increase in the quantity of JC-1 monomers (green fluorescence) in the HK-2 cells (Figure 8B), when compared to the DSPE-PEG-C60/NCTD micelle treatment group (Figure 8D). This observation suggests a significant mitochondrial damage in the HK-2 cells exposed to free NCTD.

With respect to the HepG2 cells, the diminished orange fluorescence and the increased green fluorescent intensity of the DSPE-PEG-C60/NCTD micelles, in comparison with the control group and the NCTD-treated group, indicated the collapse of the mitochondrial membrane potential, as shown in Figure 8H.

### 2.8. Cell Apoptosis by Flow Cytometry

**Figure 9:**
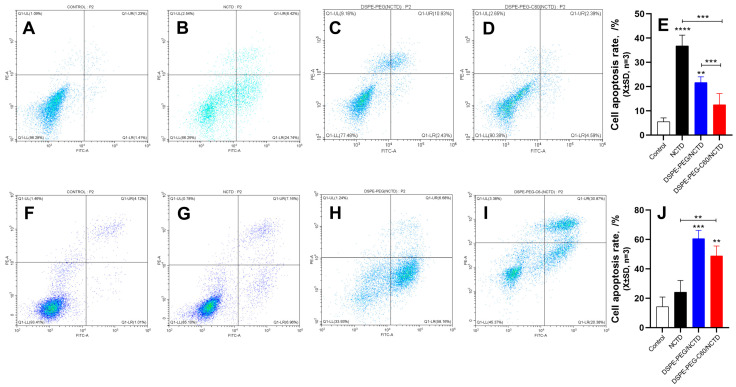
Effects of micelles and free NCTD on apoptosis of HK-2 cells and HepG2 cells: control (**A**), free NCTD (**B**), DSPE-PEG/NCTD (**C**) and DSPE-PEG-C60/NCTD (**D**). Cells were treated with micelles or free NCTD at equivalent concentrations of 75 μM NCTD (for HK-2 cells) or 150 μM NCTD (for HepG2 cells) for 24 h. Afterward, Annexin V/PI staining and flow cytometry detection were performed. (**A**–**D**) Representative pseudo-color plots of Annexin V/PI staining of HK-2 cells. (**E**) Apoptosis ratio of the calculated HK-2 cells. (**F**–**I**) Representative pseudo-color plots of Annexin V/PI staining of HepG2 cells. (**J**) Apoptosis ratio of the calculated HepG2 cells. The values presented are the means ± standard deviations of three independent experiments (** *p* < 0.01, *** *p* < 0.001 and **** *p* < 0.0001). Different colored dots represent the number of cells.

The impact of the DSPE-PEG-C60/NCTD micelles on cellular apoptosis was also assessed using flow cytometry and the Annexin V-FITC/PI double-staining method. As shown in the representative pseudo-color plots and the apoptosis ratio of the cells (Figure 9A–E), the average apoptosis rates of the HK-2 cells was 5% (control), 36% (NCTD group), 21% (DSPE-PEG/NCTD group) and 12% (DSPE-PEG-C60/NCTD group) at a 75 μM NCTD concentration. The DSPE-PEG-C60 micelles demonstrated a significant inhibitory effect on the toxicity induced by NCTD, leading to a notable decrease in the cell apoptosis ratio. This study provided additional evidence to support the notion that incorporating C60(OH)_22_ into the drug delivery system resulted in a notable decrease in the toxicity of NCTD in the HK-2normal cell line.

The HepG2 cells were also subjected to apoptosis detection to examine whether the cytotoxicity of the DSPE-PEG-C60/NCTD micelles against tumor cells was increased, as observed in the results of the cytotoxicity examination. The representative pseudo-color plots and the apoptosis ratio of the cells indicated that the NCTD-loaded DSPE-PEG-C60 micelles had an increased apoptosis ratio of approximately 48% at a concentration of 150 μM NCTD, compared with 24% in the free NCTD group (Figure 9F–J).

### 2.9. Intracellular ROS Level Evaluation

**Figure 10:**
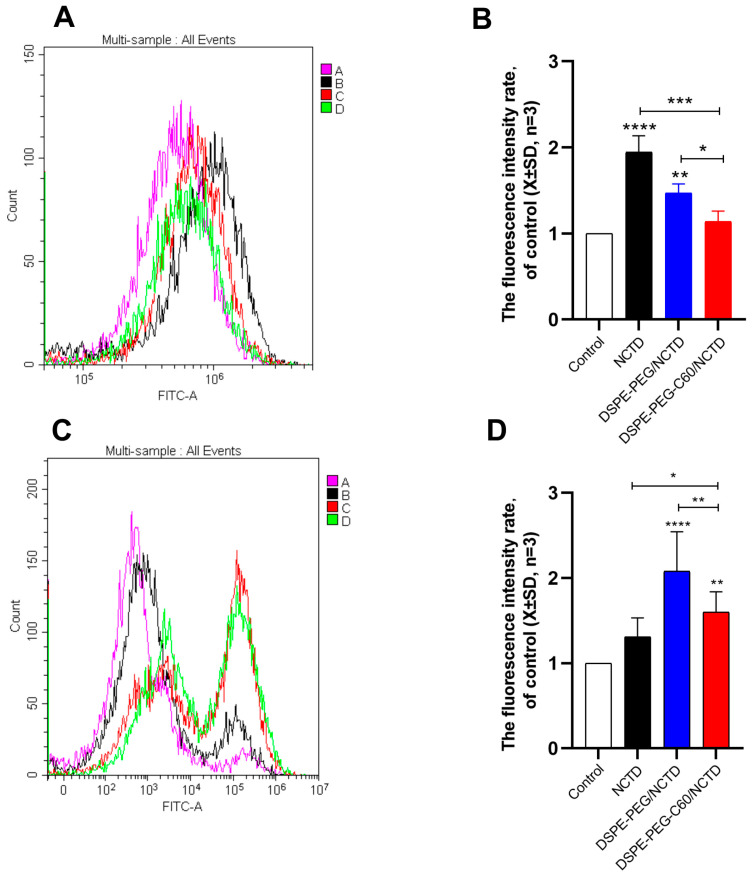
Effects of micelles and free NCTD on ROS levels in HK-2 cells and HepG2 cells: control (**A**), free NCTD (**B**), DSPE-PEG/NCTD (**C**) and DSPE-PEG-C60/NCTD (**D**). Cells were treated with micelles or free NCTD at equivalent concentrations of 75 μM NCTD (HK-2 cells) or 150 μM NCTD (HepG2 cells) for 24 h, followed by DCFDA staining and flow cytometry detection. (**A**) Representative fluorescence intensity plots of DCF in HK-2 cells. (**B**) Fluorescence intensity rate of the calculated HK-2 cells. (**C**) Representative fluorescence intensity plots of DCF in HepG2 cells. (**D**) Fluorescence intensity rate of the calculated HepG2 cells. The values presented are the means ± standard deviations of three independent experiments (* *p* < 0.05, ** *p* < 0.01, *** *p* < 0.001 and **** *p* < 0.0001).

The ROS levels in the cell lines treated with the micelles or free NCTD were assessed by flow cytometry with DCFDA staining. The NCTD-induced oxidative stress in the HK-2 cells resulted in an increased ROS level with a 1.9-fold increase in DCF fluorescence compared with the untreated cells at a 75 μM NCTD concentration. And the DCF level of the DSPE-PEG-C60 micelle group was 1.14-fold compared to the control group, which was lower than that of the free NCTD group and similar to that of the control group (Figure 10A,B). The results indicated the strong capability of C60(OH)_22_ to reduce oxidative stress in the HK-2 cells.

On the contrary, the DCF fluorescence level of the DSPE-PEG-C60/NCTD micelle group was 1.6 times that of the control group, more than that of the free NCTD group in the HepG2 cells at a 150 μM NCTD concentration (Figure 10C,D). The result confirmed the increased cytotoxicity of the NCTD-loaded micelles against the HepG2 cells.

## 3. Discussion

The physicochemical characteristics of micelles, including particle size, shape and surface charge, are important for their fate in vivo. Nanoparticles within the range of 100–200 nm in diameter have the ability to infiltrate and amass at tumor locations. Micelles were prepared with an approximate size of 100 nm in this study. These micelles exhibited a negative charge in the physiological environment due to the introduction of C60(OH)_22_. This negative charge has the potential to enhance stability by inhibiting the interaction between micelles and negatively charged vascular endothelial cells or plasma components in vivo [35]. PEG was utilized to create a compact coating on the micelles’ surface, with the purpose of prolonging the circulation time of the micelles and delaying their phagocytic clearance [36].

Although the EPR effect of nanomedicines on solid tumors is widely acknowledged, the increase in nanomedicine exposure in tumor tissue is only around 20% to 30% compared to normal tissues [37,38]. The necessity to study the reduction in severe side effects of anticancer drugs arises from the challenge of achieving complete drug enrichment in tumors. C60(OH)n has been reported as an antioxidant protector against cytotoxicity induced by chemotherapeutic drugs, as well as in other protective applications such as a radical scavenger to shield cells from radiation and as an antagonist of glutamate receptors [12,13,14,15,16]. Many studies have reported that fullerenols exhibit protective effects against doxorubicin (DOX)-induced cardiotoxicity, hepatotoxicity and nephrotoxicity in rats subjected to high doses of DOX in vivo. The cytoprotective effects of fullerenols against doxorubicin (DOX)-induced damage in normal cells in vitro were also observed in our previous studies [17]. In the present study, an investigation was conducted to examine the antioxidant properties of C60(OH)_22_ and its amphiphilic derivative DSPE-PEG-C60 in protecting normal HK-2 and L02 cells against cytotoxicity induced by NCTD. The observed decrease in cytotoxicity against the HK-2 and L02 cell lines aligns with the findings of our previous investigations. The NCTD-loaded DSPE-PEG-C60 micelles demonstrated a significant protective effect on normal cells, specifically the HK-2 cells, in comparison to the free NCTD group (with a mole ratio of NCTD:DSPE-PEG-C60 = 1:0.7). However, in vitro experiments did not reveal any protective effects of C60(OH)_22_ on the cells treated with NCTD (with a mole ratio of NCTD:C60(OH)_22_ = 1:0.7–2). One possible reason was that DSPE-PEG-C60 was more readily taken up by the cells than C60(OH)_22_.

It was also found that both of the carriers (DSPE-PEG-C60 and DSPE-PEG) enhanced the activity of NCTD on tumor cells significantly in this study. After the drugs or drug-loaded micelles reach the tumor tissue, the internalization of the drugs into the tumor cells is one of the key steps to exert the antitumor activity. The inefficient cell uptake of NCTD by tumor cells was improved by the amphiphilic carrier (DSPE-PEG or DSPE-PEG-C60).

Interestingly, in the HK-2 cells, the carriers did not increase the cytotoxicity of NCTD but decreased it (Figure 6). The microscopic fluorescence images of the AO/EB staining show that cell treatment with NCTD caused significant cell apoptosis compared to the control group. The DSPE-PEG-C60 micelles showed protection against NCTD with reduced cell apoptosis compared to the NCTD group in the HK-2 cell line (Figure 7). The loss of mitochondrial membrane potential (∆Ψ) in the cells signifies the early stage of apoptosis. The microscopic fluorescence images of JC-1 staining show that NCTD treatment caused severe cell apoptosis, indicated by the increased green fluorescence and decrease red fluorescence, compared to the control group, whose healthy cells showed red fluorescence with high mitochondrial membrane potential (ΔψM). The DSPE-PEG-C60/NCTD micelles were less potent than NCTD in damaging mitochondria, as evidenced by a relatively high ratio of red fluorescence to green fluorescence in the HK-2 cell line (Figure 8). The apoptosis examination was further validated by flow cytometry. The percentages of apoptotic cells were 36% and 12% in the NCTD and DSPE-PEG-C60/NCTD micelle groups, respectively (Figure 9). The decreased cell apoptosis of the DSPE-PEG-C60/NCTD micelles was mainly attributed to the antioxidative stress of DSPE-PEG-C60. NCTD treatment led to a 1.9-fold increase in the ROS levels, as evidenced by the increased DCF fluorescence, in comparison to the control group. In contrast, micelles modified with C60 exhibited comparable levels of ROS to the control group. These levels were significantly lower than those observed in the free NCTD group when tested on the HK-2 cell line (Figure 10).

Concurrently, the micelles loaded with NCTD demonstrated enhanced cytotoxicity against the HepG2 tumor cells. The DSPE-PEG-C60/NCTD micelles induced greater mitochondrial damage compared to the NCTD group, as demonstrated by a reduction in red fluorescence and an elevation in green fluorescence in the HepG2 cells. In the context of flow cytometry detection, it was observed that the DSPE-PEG-C60 micelles, when loaded with NCTD, exhibited a significantly higher apoptosis ratio (48%) as compared to the free NCTD group, which showed an apoptosis ratio of 24%. The elevated apoptotic ratio observed in the HepG2 cells and the decreased apoptotic ratio observed in the HK-2 cells, following the treatment with the C60-modified micelles, can be attributed to the combined effects of increased intracellular uptake facilitated by the carrier (DSPE-PEG-C60) and the interference of the antioxidant capacity of the C60 derivative with the oxidative stress induced by NCTD. Considering that hepatocellular carcinoma is the primary clinical indication for NCTD, which is mainly associated with nephrotoxicity, the utilization of the DSPE-PEG-C60/NCTD micelles could potentially provide a favorable safety profile, thus enhancing its clinical utility.

## 4. Materials and Methods

### 4.1. Materials

Norcantharidine (NCTD, Shanghai YiEn Chemical Technology Co., Ltd., Shanghai, China), Fullerenol (C60(OH)22, Hengqiu Technology Co., Ltd., Suzhou, China), DSPE-PEG (2000)-NH2 (Yuxi Pharmaceutical Technology Co., Ltd., Chongqing, China), Cell Counting Kit-8 (CCK-8, Biosharp Biology Co., Ltd., Hefei, China), Annexin V-FITC/PI Apoptosis Assay Kit (Biosharp Biology Co., Ltd., Hefei, China), Reactive Oxygen Species Assay Kit (Biosharp Biology Co., Ltd., Hefei, China), Acridine Orange and Ethidium Bromide (AO/EB, Shanghai yuanye Bio-Technology Co., Ltd., Shanghai, China), JC-1 (Biosharp Biology Co., Ltd., Hefei, China), Fetal Bovine Serum (FBS) (Gibco, California, America), DMEM/RPMI 1640/DMEM F12 Medium (Gibco, California, America) and Penicillin-streptomycin solution with double resistance (Biosharp Biology Co., Ltd., Hefei, China) were used in this study.

### 4.2. Determination of NCTD Content

The concentration of NCTD was detected using HPLC. The chromatographic conditions were as follows: column—Agilent ZORBAX ODS (4.6 × 250 nm, 5 μm); mobile phase—0.02 mol/L potassium dihydrogen phosphate solution (pH adjusted to 3.0 with phosphoric acid) and methanol in a ratio of 70:30; detection wavelength—213 nm; flow rate—1 mL/min; column temperature—35 °C; sample size—20 μL.

### 4.3. Synthesis of Fullerenol-Grafted Distearoyl Phosphatidylethanolamine—Polyethylene Glycol (DSPE-PEG-C60)

DSPE-PEG-C60 was prepared based on our previous laboratory research [17]. In short, fullerenol (C60(OH)_22_) (316 mg, 0.275 mmol) and DSPE-PEG-NH2 (500 mg, 0.275 mmol) were put in a 25 mL round-bottom flask, dissolved with 20 mL water. The solution was stirred at 60 °C for 8 h and then dialyzed with water (MW = 2000) for 6 h. After freeze-drying, brown product was obtained with a yield of about 90%.

### 4.4. Preparation of Micelles

The NCTD-loaded micelles were prepared using the ultrasonication method. NCTD (10 mg) and DSPE-PEG-C60 (or DSPE-PEG) (150 mg) were carefully weighed and put into a 50 mL centrifuge tube. Then, DMSO (1 mL) and deionized water (10 mL) were added into the centrifuge tube. After the complete dissolution of NCTD and carriers, DMSO was removed by dialysis (MW = 100). NCTD-loaded micelles were obtained by ultrasound of the dialysate followed by filtration (0.22 μm). The optimization of the preparation method, including the feed ratio, ultrasound time and ultrasound power, was investigated (Table 1).

### 4.5. Characterization of Micelles

#### 4.5.1. Morphology

The diameter and morphology of NCTD-loaded micelles were obtained using transmission electron microscopy (TEM, H-7650, Hitachi, Tokyo, Japan). The micellar solution was dropped onto a carbon-coated copper grid and allowed to dry naturally in the air. The micellar morphology was observed immediately using a transmission electron microscope. The micellar size distribution, zeta potential and PDI were determined using dynamic light scattering (DLS) with the Zetasizer Nano ZS-90 instrument (Malvern, UK).

#### 4.5.2. Determination of Entrapment Efficiency

The drug loading capacity and encapsulation efficiency were determined through ultrafiltration centrifugation. NCTD micellar solution (2 mL) was added to the upper centrifuge tube and centrifuged at 4 °C at 12,000 r/min for 20 min. The concentration of NCTD in the filtrate was determined by HPLC, obtaining the content of free NCTD in micellar solution (***W*_1_**). Afterwards, the concentration of NCTD in micellar solution was determined by HPLC, obtaining the total content of NCTD (***W*_2_**) in micelles. The mass of the carrier in the solution was ***W***_3_. The drug loading content (***LC***) and the encapsulation efficiency (***EE***) were calculated using the following formula:

$$EE=\frac{W_{2}-W_{1}}{W_{2}}\times 100\%$$

$$LC=\frac{W_{2}-W_{1}}{W_{2}+W_{3}}\times 100\%$$

#### 4.5.3. Stability Evaluation

NCTD-loaded micellar solution (10 mL) was placed in centrifuge tubes either at room temperature or at 4 °C for 0, 7, 14, 21 and 28 days. The stability of micelles system was investigated by the detection of the micelle particle size distribution (PSD), PDI and zeta potential.

#### 4.5.4. In Vitro Drug Release Assay

In order to fit the requirements of “sink condition”, SDS was added to PBS buffer solution to improve the solubility of NCTD in the buffer. NCTD-loaded micelles solution (1 mL) was put into a dialysis bag (MW = 2000), which was placed in 30 mL PBS buffer solution (containing 1% SDS) and stirred at 37 °C for 48 h at 100 r/min. The released NCTD concentration was determined at a predetermined time point using HPLC method. The release rates of NCTD from the micelles were determined by dividing the amount of NCTD released within a certain period of time by the initial NCTD content of the micelles.

### 4.6. Cell Lines and Cell Culture

Human hepatocellular carcinoma (HCC) cell lines (BEL-7402, HepG2) and immortalized normal human hepatocytes (L02) were obtained from the Cell Bank of the Chinese Academy of Sciences in Shanghai, China. Immortalized normal human kidney cells (HK-2) were purchased from Pricella Life Technology Co., Ltd. (Wuhan, China). HepG2 cells and L02 cells were cultured in DMEM medium supplemented with 10% FBS and 1% antibiotics (100 U/mL penicillin G and 0.1 mg/mL streptomycin). BEL-7402 cells were maintained in RPMI 1640 medium supplemented with 10% fetal bovine serum (FBS) and 1% antibiotics. HK-2 cells were maintained in DMEM F12 medium supplemented with 10% FBS and 1% antibiotics. Cells were maintained at 37 °C in a humidified environment with 5% CO_2_.

#### 4.6.1. Cell Viability Assay

Cell activity was detected by CCK-8 method. Logarithmic growth phase cells were seeded onto 96-well plates (3 × 10^3^/well) for 24 h and then treated either with free NCTD or with NCTD-loaded micelles at the equivalent NCTD dosage for 72 h. CCK-8 solution (10 μL) was added to each well. The cells were incubated for another 30–60 min. The absorbance value (OD value) of each hole was measured at 450 nm by MK-3 microplate reader (Thermo Fisher Scientific, United States). The untreated control samples had a default cell activity value of 100%. The cell inhibition rate was calculated using the following formula: cell inhibition rate = [(OD450 control well – OD450 administration well)/OD450 control well] × 100%.

#### 4.6.2. Acridine Orange/Ethidium Bromide (AO/EB) Staining

Cell apoptosis was detected by dual acridine orange/ethidium bromide (AO/EB) staining. Cells in the logarithmic growth phase were seeded into 24-well plates (5 × 10^4^/well), and the plates were incubated in a CO_2_ incubator (37 °C, 95% humidity and 5% CO_2_) for 24 h. Cells were treated either with free NCTD or with NCTD-loaded micelles at the same concentration of NCTD for another 24 h, followed by the digestion with trypsin and collection. Dual fluorescent staining solution (12 μL) containing acridine orange and ethidium bromide was added to each group. After a half-hour incubation, the morphology of apoptotic cells was examined using a fluorescent microscope (Nikon Eclipse Ti-S, Tokyo, Japan) at 10× magnification.

#### 4.6.3. JC-1 Staining

To measure mitochondrial membrane potential, cells at logarithmic growth stage were incubated in 24-well plates (5 × 10^4^/well) for 24 h and treated with free NCTD or NCTD-loaded micelles at the same concentration of NCTD for another 24 h. Then, the treated cells were washed and incubated with DMEM/medium containing 10 µg/mL JC-1 in the dark at 37 °C for 30 min. The cells were washed with the staining buffer, and JC-1 fluorescence was visualized using a Nikon Ti-S microscope at 20× magnification.

#### 4.6.4. Apoptosis by Flow Cytometry

The effect of NCTD-loaded micelles on cell apoptosis was also evaluated by flow cytometry. The cells in logarithmic growth stage were cultured on 6-well plates (3 × 10^5^/well) for 24 h and then treated either with free NCTD or with NCTD-loaded micelles at the same concentration of NCTD for additional 24 h. Then, the cells were collected, treated using the Annexin V-FITC/PI apoptosis assay kit and incubated in the dark at room temperature for 15 min. The stained cells were examined by flow cytometry and analyzed using the CytExpert software (Version 2.4).

#### 4.6.5. Intracellular ROS Detection

ROS production upon micelle treatment was detected by 2′,7′-dichloroflfluorescein diacetate (DCFDA) staining using a DCFDA based cell kit (Biosharp). Cells at logarithmic growth stage were cultured on 6-well plates (3 × 10^5^/well) for 24 h and treated with free NCTD and NCTD-loaded micelles respectively at the same concentration of NCTD for another 24 h. The cells were collected and stained with DCFDA in the dark at 37 °C for 30 min, which were detected by flow cytometry and analyzed using CytExpert software.

### 4.7. Statistical Analysis

All data were generated from three independent experiments and are presented as the means ± SD. The data were analyzed using Student’s *t*-test by Prism software version 8.0 (Graph Pad Software Inc., San Diego, CA, United States), and the critical level of significance was set at *p* < 0.05.

## 5. Conclusions

In this study, we prepared the DSPE-PEG-C60/NCTD micelles with an optimal particle size of 91.57 nm (PDI = 0.231) and a negative zeta potential of −13.8 mV. This study showed that the DSPE-PEG-C60/NCTD micelles decreased cytotoxicity in normal renal cells (HK-2) and increased antineoplastic activity in human hepatocellular carcinoma cells (HepG2, BEL-7402) in vitro. The scavenging capacity of the DSPE-PEG-C60 micelles against the NCTD-induced ROS showed a significant reduction in the HK-2 cells. Further investigations are in progress to examine their safety in clinically relevant animal models.

## Figures and Tables

**Table 1:** Preparation and optimization of NCTD-loaded micelles (*n* = 3).

Batch	NCTD:DSPE-PEG-C60 (*W*/*W*)	Ultrasound Time	Ultrasound Power	Particle Size (nm)	PDI	Zeta Potential (mV)
1	1:10	10 min	200 W	117.8 ± 1.041	0.375 ± 0.034	−10.1 ± 0.49
2	1:15	10 min	200 W	91.57 ± 9.78	0.231 ± 0.01	−13.8 ± 1.28
3	1:20	10 min	200 W	45.74 ± 3.35	0.439 ± 0.016	−8.07 ± 1.87
4	1:15	5 min	200 W	42.15 ± 1.13	0.428 ± 0.08	−9.25 ± 1.45
5	1:15	15 min	200 W	38.96 ± 5.37	0.295 ± 0.10	−12.0 ± 3.96
6	1:15	15 min	100 W	42.23 ± 0.845	0.430 ± 0.01	−9.73 ± 2.06
7	1:15	15 min	300 W	57.98 ± 2.35	0.260 ± 0.02	−10.4 ± 1.22

**Table 2:** Particle size distribution (PSD), zeta potential (ZP), encapsulation efficiency (EE) and drug loading content (LC) of micelles (*n* = 3).

	PSD (nm)	Zeta (mV)	EE (%)	LC (%)
DSPE-PEG/NCTD	96.1 ± 8.01	−12.0 ± 3.96	81.31 ± 0.6	5.08 ± 0.37
DSPE-PEG-C60/NCTD	91.57 ± 9.78	−13.8 ± 1.28	96.54 ± 0.03	6.05 ± 0.02

## Data Availability

No new data were created or analyzed in this study. Data sharing is not applicable to this article.

## Abbreviations

NCTD	Norcantharidin
CTD	Cantharidin
ROS	Reactive oxygen species
LD_50_	Median lethal dose
DOX	Doxorubicin hydrochloride
EPR effect	Enhanced permeability and retention effect
DSPE-PEG2000	1,2-Distearoyl-sn-glycero-3-phosphoethanolamine-N-methoxy-[poly(ethylene glycol)]; PEG MW = 2000
C60(OH)_22_	Fullerenol
FDA	Food and Drug Administration
DSPE-PEG-C60	Fullerenol-Grafted 1,2-Distearoyl-sn-glycero-3-phosphoethanolamine-N-methoxy-[poly(ethylene glycol)]
CCK-8	Cell Counting Kit-8
FITC	3′,6′-dihydroxy-5-isothiocyanato-3H-spiro[isobenzofuran-1,9′-xanthen]-3-one
PI	Propidium iodide
HPLC	High- performance liquid chromatography
DMSO	Dimethyl sulfoxide
TEM	Transmission electron microscopy
DLS	Dynamic light scattering
LC	Loading content
EE	Entrapment efficiency
SDS	Sodium dodecyl sulfate
PBS	Phosphate-buffered saline
DCFDA	2′,7′-Dichlorodihydrofluorescein diacetate
IC_50_	Half-maximal inhibitory concentration
AO and ER	Acridine orange and ethidium bromide

## References

[1] Zhou J., Ren Y., Tan L., Song X., Wang M., Li Y., Cao Z., Guo C. (2020). Norcantharidin: Research advances in pharmaceutical activities and derivatives in recent years. Biomed. Pharmacother..

[2] Pan M.S., Cao J., Fan Y.Z. (2020). Insight into norcantharidin, a small-molecule synthetic compound with potential multi-target anticancer activities. Chin. Med..

[3] Hsieh C.H., Chao K.S., Liao H.F., Chen Y.J. (2013). Norcantharidin, derivative of cantharidin, for cancer stem cells. Evid.-Based Complement. Altern. Med..

[4] Singh R., Cheng S., Li J., Kumar S., Zeng Q., Zeng Q. (2021). Norcantharidin combined with 2-deoxy-d-glucose suppresses the hepatocellular carcinoma cells proliferation and migration. 3 Biotech.

[5] Zhai B.T., Sun J., Shi Y.J., Zhang X.F., Zou J.B., Cheng J.X., Fan Y., Guo D.Y., Tian H. (2022). Review targeted drug delivery systems for norcantharidin in cancer therapy. J. Nanobiotechnol..

[6] Martínez-Razo G., Domínguez-López M.L., de la Rosa J.M., Fabila-Bustos D.A., Reyes-Maldonado E., Conde-Vázquez E., Vega-López A. (2023). Norcantharidin toxicity profile: An in vivo murine study. Naunyn Schmiedebergs Arch. Pharmacol..

[7] Chi J., Jiang Z., Chen X., Peng Y., Liu W., Han B., Han B. (2019). Studies on anti-hepatocarcinoma effect, pharmacokinetics and tissue distribution of carboxymethyl chitosan based norcantharidin conjugates. Carbohydr. Polym..

[8] Andrade E.B., Martinez A. (2017). Free radical scavenger properties of metal-fullerenes: C-60 and C-82 with Cu, Ag and Au (atoms and tetramers). Comput. Theor. Chem..

[9] Kazemzadeh H., Mozafari M. (2019). Fullerene-based delivery systems. Drug Discov. Today.

[10] Kepinska M., Kizek R., Milnerowicz H. (2018). Fullerene as a doxorubicin nanotransporter for targeted breast cancer therapy: Capillary electrophoresis analysis. Electrophoresis.

[11] Mashino T. (2022). Development of Bio-active Fullerene Derivatives Suitable for Drug. Yakugaku Zasshi.

[12] Injac R., Boskovic M., Perse M., Koprivec-Furlan E., Cerar A., Djordjevic A., Strukelj B. (2008). Acute doxorubicin nephrotoxicity in rats with malignant neoplasm can be successfully treated with fullerenol C60(OH)24 via suppression of oxidative stress. Pharmacol. Rep..

[13] Injac R., Perse M., Cerne M., Potocnik N., Radic N., Govedarica B., Djordjevic A., Cerar A., Strukelj B. (2009). Protective effects of fullerenol C60(OH)24 against doxorubicin-induced cardiotoxicity and hepatotoxicity in rats with colorectal cancer. Biomaterials.

[14] Petrovic D., Seke M., Borovic M.L., Jovic D., Borisev I., Srdjenovic B., Rakocevic Z., Pavlovic V., Djordjevic A. (2018). Hepatoprotective effect of fullerenol/doxorubicin nanocomposite in acute treatment of healthy rats. Exp. Mol. Pathol..

[15] Tang J., Zhang R., Guo M., Shao L., Liu Y., Zhao Y., Zhang S., Wu Y., Chen C. (2018). Nucleosome-inspired nanocarrier obtains encapsulation efficiency enhancement and side effects reduction in chemotherapy by using fullerenol assembled with doxorubicin. Biomaterials.

[16] Ding M., Li M., Zhang E.M., Yang H.L. (2020). FULLEROL alleviates myocardial ischemia-reperfusion injury by reducing inflammation and oxidative stress in cardiomyocytes via activating the Nrf2/HO-1 signaling pathway. Eur. Rev. Med. Pharmacol. Sci..

[17] Xu B., Ding Z., Hu Y., Zhang T., Shi S., Yu G., Qi X. (2022). Preparation and Evaluation of the Cytoprotective Activity of Micelles with DSPE-PEG-C60 as a Carrier Against Doxorubicin-Induced Cytotoxicity. Front. Pharmacol..

[18] Ghosh B., Biswas S. (2021). Polymeric micelles in cancer therapy: State of the art. J. Control. Release.

[19] Perumal S., Atchudan R., Lee W. (2022). A Review of Polymeric Micelles and Their Applications. Polymers.

[20] Gill K.K., Kaddoumi A., Nazzal S. (2015). PEG-lipid micelles as drug carriers: Physiochemical attributes, formulation principles and biological implication. J. Drug Target..

[21] Liang H., Ren X., Qian J., Zhang X., Meng L., Wang X., Li L., Fang X., Sha X. (2016). Size-Shifting Micelle Nanoclusters Based on a Cross-Linked and pH-Sensitive Framework for Enhanced Tumor Targeting and Deep Penetration Features. ACS Appl. Mater. Interfaces.

[22] Mellor R.D., Uchegbu I.F. (2022). Ultrasmall-in-Nano: Why Size Matters. Nanomaterials.

[23] Yu F., Jiang F., Tang X., Wang B. (2018). N-octyl-N-arginine-chitosan micelles for gambogic acid intravenous delivery: Characterization, cell uptake, pharmacokinetics, and biodistribution. Drug Dev. Ind. Pharm..

[24] Song Z., Deng P., Teng F., Zhou F., Zhu W., Feng R. (2017). Development on PEG-modified Poly (Amino Acid) Copolymeric Micelles for Delivery of Anticancer Drug. Anticancer Agents Med. Chem..

[25] Al-Amili M., Jin Z., Wang Z., Guo S. (2021). Self-Assembled Micelles of Amphiphilic PEGylated Drugs for Cancer Treatment. Curr. Drug Targets.

[26] Li Y., Zhang T., Liu Q., He J. (2019). PEG-Derivatized Dual-Functional Nanomicelles for Improved Cancer Therapy. Front. Pharmacol..

[27] Hwang D., Ramsey J.D., Kabanov A.V. (2020). Polymeric micelles for the delivery of poorly soluble drugs: From nanoformulation to clinical approval. Adv. Drug Deliv. Rev..

[28] Lee K.S., Chung H.C., Im S.A., Park Y.H., Kim C.S., Kim S.B., Rha S.Y., Lee M.Y., Ro J. (2008). Multicenter phase II trial of Genexol-PM, a Cremophor-free, polymeric micelle formulation of paclitaxel, in patients with metastatic breast cancer. Breast Cancer Res. Treat..

[29] Kumbar V.M., Muddapur U., Bin Muhsinah A., Alshehri S.A., Alshahrani M.M., Almazni I.A., Kugaji M.S., Bhat K., Peram M.R., Mahnashi M.H. (2022). Curcumin-Encapsulated Nanomicelles Improve Cellular Uptake and Cytotoxicity in Cisplatin-Resistant Human Oral Cancer Cells. J. Funct. Biomater..

[30] Kulthe S.S., Choudhari Y.M., Inamdar N.N., Mourya V. (2012). Polymeric micelles: Authoritative aspects for drug delivery. Des. Monomers Polym..

[31] Li W., Wu J., Zhang J., Wang J., Xiang D., Luo S., Li J., Liu X. (2018). Puerarin-loaded PEG-PE micelles with enhanced anti-apoptotic effect and better pharmacokinetic profile. Drug Deliv..

[32] Demina T., Grozdova I., Krylova O., Zhirnov A., Istratov V., Frey H., Kautz H., Melik-Nubarov N. (2005). Relationship between the structure of amphiphilic copolymers and their ability to disturb lipid bilayers. Biochemistry.

[33] Barron A.R. (2016). [60]Fullerene-peptides: Bio-nano conjugates with structural and chemical diversity. J. Enzym. Inhib. Med. Chem..

[34] Zhang L.W., Yang J., Barron A.R., Monteiro-Riviere N.A. (2009). Endocytic mechanisms and toxicity of a functionalized fullerene in human cells. Toxicol. Lett..

[35] Ilinskaya A.N., Shah A., Enciso A.E., Chan K.C., Kaczmarczyk J.A., Blonder J., Simanek E.E., Dobrovolskaia M.A. (2019). Nanoparticle physicochemical properties determine the activation of intracellular complement. Nanomed. Nanotechnol. Biol. Med..

[36] Choi K.Y., Min K.H., Yoon H.Y., Kim K., Park J.H., Kwon I.C., Choi K., Jeong S.Y. (2011). PEGylation of hyaluronic acid nanoparticles improves tumor targetability in vivo. Biomaterials.

[37] Kobayashi H., Choyke P.L. (2016). Super enhanced permeability and retention (SUPR) effects in tumors following near infrared photoimmunotherapy. Nanoscale.

[38] Attia M.F., Anton N., Wallyn J., Omran Z., Vandamme T.F. (2019). An overview of active and passive targeting strategies to improve the nanocarriers efficiency to tumour sites. J. Pharm. Pharmacol..

